# Application of the protection motivation theory to understand determinants of compliance with the measure of banning gathering size >4 in all public areas for controlling COVID-19 in a Hong Kong Chinese adult general population

**DOI:** 10.1371/journal.pone.0268336

**Published:** 2022-05-10

**Authors:** Yanqiu Yu, Mason M. C. Lau, Joseph T. F. Lau

**Affiliations:** 1 Centre for Health Behaviours Research, Jockey Club School of Public Health and Primary Care, The Chinese University of Hong Kong, Hong Kong, China; 2 School of Psychiatry, Wenzhou Medical University, Wenzhou, China; Iowa State University, UNITED STATES

## Abstract

**Background:**

Restriction of gathering size in all public areas is a newly and commonly exercised governmental social distancing policy during the COVID-19 pandemic. Its effectiveness depends on the general public’s compliance. This study applied the Protection Motivation Theory (PMT) to investigate determinants (i.e., perceived severity, perceived susceptibility, perceived response efficacy, and perceived self-efficacy) of compliance with the social distancing policy of banning gathering of >4 people in all public areas (BG4PA) in the Hong Kong general adult population.

**Methods:**

300 participants were interviewed through a population-based telephone survey during April 21–28, 2020.

**Results:**

The compliance rate of BG4PA was high (78%). Adjusted for the background factors, multiple linear regression analysis found that perceived response efficacy and perceived self-efficacy were significantly and positively associated with compliance with BG4PA (*p*<0.05), while the associations between perceived severity/perceived susceptibility and compliance were of marginal significance (0.05<*p*<0.10). A stepwise linear regression model considered four PMT constructs as candidates; its final model only selected self-efficacy but not the other three PMT constructs.

**Conclusions:**

PMT can be applied to understand compliance with BG4PA. Perceived response efficacy and perceived self-efficacy were more influential than perceived severity and perceived susceptibility. Health promotion may focus on improving coping appraisal.

## Introduction

Social distancing is an effective and commonly adopted public health measure that can be used to contain the transmission of emerging respiratory infectious diseases, such as severe acute respiratory syndrome (SARS) and swine flu (H1N1), via reduction of close physical contact with other people [1]. In the presence of asymptomatic transmissions [2], various governments have exercised social distancing measures during the COVID-19 pandemic [3, 4]. There were 8,505,042 COIVD-19 cases and 456,973 deaths reported in over 200 countries (as of June 20, 2020) [5]. Some of the social distancing measures (e.g., closure of schools and cancellation of public events) are conventional. Yet, the strict legal measure to restrict gathering size in all public areas within a city or country (e.g., not >4) is a new public health measure. It deserves a better understanding for various reasons. First, it is a potentially effective control measure, as it can effectively reduce the transmission rate of COVID-19 [6]. Second, some people showed low adherence to such a measure [7], possibly due to its comprehensive and tremendous impacts on daily lives (e.g., social activities, work, entertainment, religious activities, and even political protests). Third, it is a controversial measure that had been used in a growing number of countries (e.g., the U.K. [8] and Germany [9]) but had also lead to massive protests in some countries (e.g., the U.S.) [10].

The present study was conducted in Hong Kong, where the number of reported COVID-19 cases was relatively low (1,129 cases and five deaths as of June 21, 2020), while 61.2% of such cases were believed to be imported from other countries [11]. In addition to other social distancing measures (e.g., mandatory quarantine for travelers from other countries/regions and class suspension), the Hong Kong government implemented the measure of banning gathering of >4 people in all public areas (BG4PA) from March 29 to May 4, 2020, during which the present study was conducted (from April 21 to 28, 2020). The gathering size was later increased to >8 people (from May 5 to June 18, 2020), and then >50 people (from June 19 to July 2, 2020) [12]. In Hong Kong, the fine for violation was HKD 2,000 (around USD 250); more than 700 people have been fined for violating the measure [13].

Like other public health measures, the effectiveness of BG4PA depends on the general public’s compliance. Comparisons of the compliance rates across countries, together with modeling based on such data and other social distancing indicators, may partially explain the global variations of the severity of COVID-19. Health promotion has improved compliance rates of public health measures unrelated to COVID-19, such as tobacco-free campus policy in the U.S. [14] and hand hygiene in patient care [15]. Identification of modifiable determinants may improve the effectiveness of health promotion efforts. Existing studies have reported significant factors of compliance with national public health policies concerning COVID-19, including socio-demographics (e.g., female), perceptions related to COVID-19 (e.g., perceived risk of COVID-19 infection), fear of COVID-19, and trust in government or healthcare system [16–21]. Implications of these studies may, however, be rather limited, as a wide range of social distancing behaviors (e.g., avoiding going out versus avoiding social gatherings) were combined in the outcome variables, whereas factors of specific social distancing behaviors may vary. Identification of factors of specific social distancing measures (e.g., BG4PA) may facilitate health promotion of the corresponding measures. To our knowledge, no study has looked at the factors of the specific social distancing measure of restricting gathering size in public areas.

Application of behavioral theories to understand social distancing behaviors is warranted, as theory-based interventions are more likely to be effective [22]. Theory-based studies are potentially comparable across countries and can inform the improvement of the theory of concern. The Protection Motivation Theory (PMT) of fear appeals postulates that threat (fear) appraisal and coping appraisal may induce protection motivation that would increase or reduce health-related behaviors to avoid the potential threats [23]. While threat appraisal (perceived severity and perceived susceptibility) assesses how serious the disease would be and how likely one would contract the disease, coping appraisal assesses one’s expected efficacy that performance of the recommended behavior can reduce/remove the threat (i.e., perceived response efficacy) and one’s ability in successfully executing the recommended behavior (i.e., perceived self-efficacy).

The PMT has been applied to investigate determinants of the uptake of personal preventive behaviors related to H1N1 [24, 25] and intention to receive influenza/COVID-19 vaccination. Since its constructs are highly relevant to the social distancing measures related to the COVID-19 pandemic, PMT provides a potentially useful theoretical framework to understand the determinants of compliance with the gathering-size policy regarding COVID-19. One empirical study conducted among Iranian health workers found that perceived response efficacy and self-efficacy of preventive measures were positively associated with the protection motivation to perform COVID-19 preventive behaviors, which was in turn significantly associated with the actual behaviors (e.g., wearing gloves/face-masks and handwashing) [26]. Another study found that perceived response efficacy and perceived self-efficacy (but not perceived severity and perceived susceptibility) were positively associated with the intention to engage in some social distancing related behaviors (e.g., ordered food instead of dining in the common area and avoided common restroom) among university students during the COVID-19 outbreak in Malaysia [27]. To our knowledge, no study has applied the PMT to understand governmental social distancing policies, including gathering size measures, during the COVID-19 pandemic in the general population. The present study investigated the levels and associated factors derived from the PMT (i.e., perceived severity, perceived susceptibility, perceived response efficacy, and perceived self-efficacy) of compliance with the specific governmental social distancing policy of BG4PA in the Hong Kong general adult population.

## Methods

### Participants and data collection

A random telephone survey was conducted among Hong Kong Chinese adults (aged ≥18 years) during April 21–28, 2020; interviews were made from 6 pm to 10:30 pm (10 to 15 minutes) by some experienced interviewers to avoid over-sampling non-working individuals. 500,000 household telephone numbers were randomly drawn from the updated landline telephone directories to be used as seed numbers. To further solicit potentially unlisted numbers, each listed number was paired with three extra ‘phone’ numbers by randomizing the last two digits of each listed number. These generated numbers, after removing duplications, were mixed with the original 500,000 listed numbers, which comprised the sampling frame. Random numbers were drawn from this sampling frame for interviews; invalid numbers (e.g., commercial numbers and fax numbers) were replaced by additional random numbers. The household member whose birthday was closest to the interview date was invited to join the study. Unanswered telephone calls were given at least three attempts before being classified as invalid. Unavailable eligible participants were contacted again by appointments. No incentives were given to the participants. Verbal informed consent was obtained from the participants and the ethics approval was obtained from the Survey and Behavioral Research Ethics Committee of the corresponding author’s affiliated institution (No. SBRE-19-661).

Excluding invalid numbers involving non-households and empty numbers, 552 random numbers were able to identify eligible prospective participants in households, while 366 called households that failed to identify an eligible person (119 ineligible cases and 247 unknown eligibilities due to immediate refusal). The response rate, defined as the number of completed interviews ÷ the number of eligible contacts, was 54.3% (i.e., 300 ÷ 552 × 100% = 54.3%).

### Measures

#### Background variables

Information was collected about sex, age, current marital/cohabitation status, and educational level among participants.

#### Compliance with BG4PA

The item was: “How often were you able to avoid gathering with >4 people in public areas in the past week?” (1 = never to 5 = always).

### PMT constructs

1. Perceived severity: The single item was “How much would the COVID-19 affect your life if you contracted COVID-19?” (0 = no impacts to 10 = extremely severe impacts). The question was extracted from the Brief Illness Perception Questionnaire and has been used as a single item in various studies [28, 29].
2. Perceived susceptibility: The single item was “What is the probability that you or your family members would contract COVID-19?” (1 = extremely low to 5 = extremely high). The single item question has also been used in a number of previous studies [30, 31].
3. Perceived response efficacy: The single item was “How effective do you think is the governmental measure of BG4PA?” (1 = extremely low effectiveness to 5 = extremely high effectiveness).
4. Perceived self-efficacy: The single item was: “To what degree you are able to comply with the governmental preventive measure and avoid gatherings of >4 persons in public areas if you wanted to do so?” (1 = extremely low to 10 = extremely high).

### Statistical analysis

The software PASS 11.0 was used to conduct sample size planning for correlation coefficients (*r*) describing the strength of the relationship between two variables [32]. Assuming a power of 0.80 (i.e., the probability of detecting a statistically significant correlation if there is a true one) and alpha of 0.05 (i.e., the significance level, two-tailed), the sample size of 300 would have the smallest detectable *r* of 0.16, which represents a small effect size [33]. Thus, this study would have a good statistical power of at least 0.80 for detecting the correlations with *r* ≥0.16. The sample size was deemed to be adequate.

Pearson correlation coefficients were derived for the correlations among the four PMT constructs. Simple linear regression analyses were performed to investigate the associations between the background factors/PMT constructs and the dependent variable of compliance with BG4PA. Multiple linear regression analysis was performed to investigate the individual associations between the PMT constructs and the compliance outcome, adjusted for background factors. A stepwise regression procedure entering all the four PMT constructs in one model was performed to extract potential independent factors of compliance with BG4PA. Standardized coefficients (*β*) were reported in this report. The statistical analyses were performed by using SPSS 21.0. *p*<0.05 and 0.05<*p*<0.10 were defined as statistically significant and marginally significant, respectively (two-tailed).

## Results

### Descriptive statistics

The participants’ socio-demographic characteristics are described in Table 1. The prevalence of compliance with BG4PA (frequently/always) was 78.0%. The mean (SD; range) of the compliance outcome was 4.1 (1.1; 1–5). The mean (SD; range) of perceived severity, perceived susceptibility, perceived response efficacy, and perceived self-efficacy were 8.3 (1.8; 0–10), 2.5 (0.9; 1–5), 3.8 (1.0; 1–5), and 8.6 (1.7; 1–10), respectively.

**Table 1:** Descriptive statistics of the participants (n = 300).

	n	%
**Background factors**		
Sex		
Male	98	32.7
Female	202	67.3
Age		
18–35	53	17.7
36–55	102	34.0
56–65	65	21.7
>65	77	25.7
Missing data	3	1.0
Current marital/cohabitation status		
Single/separated/divorced/widow/widower	104	34.7
Cohabitation/married	196	65.3
Educational level		
≤Primary school	53	17.7
Middle school/matriculation	169	56.3
≥College	77	25.6
Missing data	1	0.3
**Compliance with banning gatherings of >4 people in all public areas (BG4PA)**		
Never	18	6.0
Rarely	9	3.0
Sometimes	39	13.0
Frequently	81	27.0
Always	153	51.0

### Correlations among the PMT constructs

Three such significant correlations were found (Table 2): 1) Perceived severity was positively correlated with perceived susceptibility (*r* = 0.21; *p*<0.001). 2) Perceived susceptibility was negatively correlated with perceived response efficacy (*r* = −0.12; *p* = 0.042). 3) Perceived response efficacy was positively correlated with perceived self-efficacy (*r* = 0.22; *p*<0.001). The correlation between perceived severity and perceived self-efficacy was of marginal significance (*r* = 0.11; *p* = 0.062). The other correlations (those between perceived severity and perceived response efficacy and between perceived susceptibility and perceived self-efficacy) were statistically non-significant.

**Table 2:** Pearson correlation coefficients among the constructs of the protection motivation theory.

	1	2	3	4
**Threat appraisal variables**				
1. Perceived severity	-			
2. Perceived susceptibility	0.21 (*p*<0.001)	-		
**Coping appraisal variables**				
3. Perceived response efficacy	0.05 (*p* = 0.439)	−0.12 (*p* = 0.042)	-	
4. Perceived self-efficacy	0.11 (*p* = 0.062)	−0.06 (*p* = 0.341)	0.22 (*p*<0.001)	-

### Factors of compliance with BG4PA

#### Background factors

Simple linear regression models showed that participants aged 56–65 years (*β* = 0.20; *p* = 0.006) and those who had received tertiary education or above (*β* = 0.16; *p* = 0.036) were more likely than others to comply with BG4PA. The associations between sex/current marital status and compliance were statistically non-significant (Table 3). A multiple linear regression model entering all the above background factors in one model reported consistent results regarding the direction and significance of the associations; such results were not tabulated.

**Table 3:** Simple linear regression analysis on the background factors of compliance with BG4PA (n = 300).

	Compliance with banning gathering of >4 people in all public areas (BG4PA)	
	*β* ^*a*^	*p*
Sex		
Male		
Female	0.03	0.609
Age		
18–35		
36–55	0.04	0.657
56–65	0.20	0.006
>65	0.07	0.387
Current marital/cohabitation status		
Single/separated/divorced/widow/widower		
Cohabitation/married	0.02	0.704
Educational level		
≤Primary school		
Middle school/matriculation	0.04	0.611
≥College	0.16	0.036

^a^ Standardized coefficients were reported.

#### Associations between the PMT constructs and compliance with BG4PA

The results of the simple regression models are summarized in Table 4. Adjusted for the background factors, perceived response efficacy (*β* = 0.16; *p* = 0.009) and perceived self-efficacy (*β* = 0.53; *p*<0.001) were positively associated with compliance with BG4PA, while the associations between perceived severity (*β* = 0.10; *p* = 0.080)/perceived susceptibility (*β* = −0.11; *p* = 0.062) and compliance were of marginal statistical significance (0.05<*p*<0.10). The stepwise linear regression model using the four PMT constructs as candidates (adjusted for background factors) only selected perceived self-efficacy in its final model (*β* = 0.53; *p*<0.001). Notably, the directions and significance of the associations between background factors and compliance with BG4PA in the above models were highly consistent with those in Table 3.

**Table 4:** Associations between the constructs of the Protection Motivation Theory (PMT) and compliance with BG4PA (n = 300).

	Compliance with banning gathering of >4 people in all public areas (BG4PA)								
	Simple linear regression			Adjusted linear regression ^a^			Stepwise linear regression ^b^		
	*β* ^c^	*p*	*R* ^*2*^	*β* ^c^	*p*	*R* ^*2*^	*β* ^c^	*p*	*R* ^*2*^
**Threat appraisal variables**									
Perceived severity	0.12	0.032	0.01	0.10	0.080	0.08	-	-	-
Perceived susceptibility	−0.11	0.054	0.01	−0.11	0.062	0.08	-	-	-
**Coping appraisal variables**									
Perceived response efficacy	0.15	0.009	0.02	0.16	0.009	0.09	-	-	-
Perceived self-efficacy	0.55	<0.001	0.30	0.53	<0.001	0.32	0.53	<0.001	0.32

^a^ Adjusted linear regression were conducted to test the individual associations between the four PMT constructs and compliance with BG4PA, after adjusting for background factors, including sex, age, current marital/cohabitation status, and educational level.

^b^Stepwise linear regression was conducted by entering all the four PMT constructs in one model and using stepwise as the variable selection method, after adjusted for the background factors, including sex, age, current marital/cohabitation status, and educational level.

^c^Standardized coefficients were reported.

## Discussion

It is encouraging to observe a high compliance rate of BG4PA (close to 80% endorsed the ‘frequently/always’ responses), although Hong Kong is densely populated and gathering of >4 people in public areas used to be very common. Comparatively, a pre-print study claimed a much lower compliance rate (60.2%) for the general social distancing policies implemented in the U.S. [34]. There are a few contextual reasons behind the high compliance rate in Hong Kong. First, the Chinese culture in Hong Kong emphasized less personal freedom and more collective good [35], so people might be more willing to exchange personal inconvenience for successful control of the pandemic. Second, the SARS experience in Hong Kong may have drilled people to comply with public health measures against emerging respiratory diseases. For instance, facemask wearing and frequent handwashing were almost universal (>95%) in the Hong Kong general population [36, 37]. Third, people in Hong Kong tended to take COVID-19 seriously since its initial outbreak in mainland China. School and office closure occurred as early as January 2020; this study also found a high level of perceived severity (mean of 8.3 out of a range of 0–10). Lower compliance rates were found among those with lower educational levels. The literature has found a disproportionately high prevalence of confirmed COVID-19 cases among socially disadvantaged groups [38, 39]. The social disparity in prevention in the context of COVID-19 has also been found. A study reported that disadvantaged social groups were less informed and possessed fewer resources for the prevention of COVID-19 [40]. It is an important topic that requires further exploration.

The adjusted analysis found that perceived response efficacy and perceived self-efficacy were positively associated with compliance with BG4PA. The significant associations corroborate a previous study that applied PMT to understand the intention of performing social distancing behavior among university students in Malaysia [27]. In addition, in the present study, those with higher levels of perceived severity related to COVID-19 showed higher compliance rates; the association reached marginal significance; it might have been statistically significant given a slightly larger sample size. Perceived severity related to a disease is also a construct of the Health Belief Model [41]. It has been found to be significantly associated with many health-related behaviors, including those related to preventive behaviors taken up during the SARS period [42] and the COVID-19 pandemic (a preprint) [43]. This study, however, found an ‘unexpected’ negative association between perceived susceptibility and compliance, possibly because of the cross-sectional study design. Instead of the postulation that higher perceived susceptibility would induce protection motivation, it is plausible that the reverse had occurred, i.e., those who practiced social distancing would perceive weaker susceptibility to contracting the virus. Previous studies also argued that perceived susceptibility might not be a key factor of compliance with preventive behaviors when a community outbreak was not severe [17], which was the case in Hong Kong. Furthermore, it is possible that perceived susceptibility that was positively related to fear would cause irrational thoughts, which would lead to a low level of compliance [44]. Longitudinal research is thus warranted to discern causality between these two variables. Overall, it seems that in the case of compliance with the specific governmental social distancing measures, the coping appraisal might be more important than threat appraisal. The contention aligned with the findings of the aforementioned study among Malaysian university students [27]. Furthermore, it is plausible that perceived severity/susceptibility would affect compliance only if people had perceived response efficacy and/or perceived self-efficacy. Such moderation hypotheses should be tested in the future.

The cognitive emotional regulation theory suggests that an external stimulus (e.g., the COVID-19 pandemic) may result in cognitive bias [45], which may cause some people to perceive various aspects of social distancing negatively. Accordingly, it is understandable that perceived self-efficacy was correlated with other PMT constructs (perceived response efficacy and perceived severity). Given such significant (and marginally significant) associations, the other three PMT constructs were not selected by the stepwise regression model that had already selected perceived self-efficacy. Thus, perceived self-efficacy seems to be more important than the other three constructs of the PMT in influencing compliance with BG4PA. Upon confirmation of this finding in future studies, health promotion is warranted to improve compliance with BG4PA and other social distancing measures through the improvement of self-efficacy. The literature shows that in general interventions that focused on performance accomplishment, verbal persuasion, physiological arousal, and vicarious experience were able to increase self-efficacy [46]. Perceived response efficacy, which is another potentially important determinant of compliance with BG4PA, can be enhanced by building up trust in the government and reducing worries/fears about COVID-19 [47, 48]. Future pilot studies are warranted to evaluate the efficacy of such interventions.

The present study has several limitations. First, as mentioned, the study’s sample size and hence the statistical power was relatively small; some marginally significant associations could have been significant given a larger sample size. Nonetheless, numerous local random telephone surveys reported comparable sample sizes [49–51]. It should be noted that those who did not complete the interviews were completely excluded from this study, the characteristics between the participants and non-participants hence could not be compared. Furthermore, the study sample may be slightly overrepresented by females, although its age distribution and educational levels were similar to those of the 2019 Hong Kong census data [52]. Second, there was potential social desirability bias regarding self-reported compliance with BG4PA. Third, we are unable to make causal and temporal inferences due to the use of a cross-sectional study design; longitudinal studies are needed. Fourth, some studies, but not ours, have included the extended PMT construct of response cost, which is less directly applicable to the present context as the cost of compliance was hard to quantify. In the absence of validated scales, it is a limitation that single items were used as independent variables of this study, although some of these items have been used in other related published studies. As various countries differed in the content (e.g., fines), enforcement, and background of their social distancing policies, caution is needed for the generalization of the findings to other countries. Last but not least, as the policy and information about the COVID-19 pandemic kept changing, it is unknown whether the identified factors would be consistent over time and stages of the pandemic.

## Conclusions

To conclude, the present study revealed a relatively high level of compliance with BG4PA in the adult general population in Hong Kong, China. The PMT was in general applicable to understand the general public’s compliance with this social distancing policy. Future research may look at its applicability to other social distancing measures. Coping appraisal, especially perceived self-efficacy, was potentially more influential to compliance with BG4PA than threat appraisal. The findings have implications for other countries that had adopted similar social distancing policies. Future longitudinal studies are warranted to confirm the applicability of PMT in such countries. Such studies may also test other theories to make comparisons with the PMT. The restriction of gathering size in public areas during the COVID-19 pandemic is an important new public health measure that requires future research.

## Supporting information

S1 File Anonymized dataset (n = 300). (SAV)
